# Long-term survival and quality of life after intensive care for patients 80 years of age or older

**DOI:** 10.1186/s13613-015-0053-0

**Published:** 2015-06-03

**Authors:** Finn H Andersen, Hans Flaatten, Pål Klepstad, Ulla Romild, Reidar Kvåle

**Affiliations:** Department of Anesthesia and Intensive Care, Møre and Romsdal Health Trust, Ålesund Hospital, 6026 Ålesund, Norway; Department of Circulation and Medical Imaging, Faculty of Medicine, Norwegian University of Science and Technology, Pb. 8905, 7491 Trondheim, Norway; Department of Anesthesia and Intensive Care, Haukeland University Hospital, Jonas Lies vei 65, 5021 Bergen, Norway; University of Bergen, Pb. 7800, 5200 Bergen, Norway; Department of Anesthesia and Intensive Care, St. Olavs Hospital, Prinsesse Kristinas gate 3, 7030 Trondheim, Norway; Nord-Trøndelag Health Trust, Pb. 333, 7601 Levanger, Norway; The Public Health Agency of Sweden, 831 40 Østersund, Sweden

**Keywords:** Intensive care unit, Elderly, Octogenarians, Survival, Mortality, HRQOL, Long-term outcome

## Abstract

**Background:**

Comparison of survival and quality of life in a mixed ICU population of patients 80 years of age or older with a matched segment of the general population.

**Methods:**

We retrospectively analyzed survival of ICU patients ≥80 years admitted to the Haukeland University Hospital in 2000–2012. We prospectively used the EuroQol-5D to compare the health-related quality of life (HRQOL) between survivors at follow-up and an age- and gender-matched general population. Follow-up was 1–13.8 years.

**Results:**

The included 395 patients (mean age 83.8 years, 61.0 % males) showed an overall survival of 75.9 (ICU), 59.5 (hospital), and 42.0 % 1 year after the ICU. High ICU mortality was predicted by age, mechanical ventilator support, SAPS II, maximum SOFA, and multitrauma with head injury. High hospital mortality was predicted by an unplanned surgical admission. One-year mortality was predicted by respiratory failure and isolated head injury. We found no differences in HRQOL at follow-up between survivors (*n* = 58) and control subjects (*n* = 179) or between admission categories. Of the ICU non-survivors, 63.2 % died within 2 days after ICU admission (*n* = 60), and 68.3 % of these had life-sustaining treatment (LST) limitations. LST limitations were applied for 71.3 % (*n* = 114) of the hospital non-survivors (ICU 70.5 % (*n* = 67); post-ICU 72.3 % (*n* = 47)).

**Conclusions:**

Overall 1-year survival was 42.0 %. Survival rates beyond that were comparable to those of the general octogenarian population. Among survivors at follow-up, HRQOL was comparable to that of the age- and sex-matched general population. Patients admitted for planned surgery had better short- and long-term survival rates than those admitted for medical reasons or unplanned surgery for 3 years after ICU admittance. The majority of the ICU non-survivors died within 2 days, and most of these had LST limitation decisions.

**Electronic supplementary material:**

The online version of this article (doi:10.1186/s13613-015-0053-0) contains supplementary material, which is available to authorized users.

## Background

In many countries, aged populations may increase by 40–50 % in the coming decades [1–3]. A similar increase is expected in the proportion of older patients admitted to intensive care units (ICU). Patients 80 years of age or older currently constitute between 8.9 and 13.8 % of large national ICU registries [4–7]. Australia and New Zealand showed 5.6 % annual increases in the numbers of octogenarians that entered the ICU [4]; in Denmark, an 18 % increase was observed from 2005 to 2011 [5].

Few recent studies have focused on long-term health-related quality of life (HRQOL) in aged ICU survivors, and even fewer have compared octogenarian ICU patients to an older segment of the general population. These studies were mainly performed in medical ICUs and included small sample sizes, due to high short-term mortality [8, 9]. One- and 2-year mortalities in octogenarians are reported to be as high as 72.0 and 79 % [9], respectively. Thus, it is important to identify factors among the older population that predict benefit from ICU treatment, establish prognostic factors for long-term survival, and elucidate the HRQOL.

This study aimed to:

1. Compare survival and HRQOL between older patients and age-matched control groups from the general population;
2. Identify predictors for short- and long-term mortality among older ICU patients; and
3. Compare survival and HRQOL scores between the different SAPS II admission categories: admissions for planned surgery, unplanned surgery, and medical reasons.

## Methods

Haukeland University Hospital is a tertiary university hospital in Bergen, Norway, which serves approximately one million inhabitants. The general ICU has ten beds (burn, cardiac surgery, coronary, and neonatal units are separate units, and are not included in this study). The annual number of admissions is about 500, and 7–8 % of patients are 80 years of age or older. There were no large changes in practice or organization of the ICU during the study period besides general development in medicine and intensive care.

### Study design

The first part of this study was a retrospective analysis of patients ≥80 years old, which were admitted to this general ICU between the 1st of January 2000 and the 31st of December 2012. These data were extracted from the dedicated ICU database with daily, prospectively collected data. Re-admissions, non-Norwegian patients, and admissions with errors in patient ID were excluded. For all included patients, we assessed the following:

1. Age and gender;
2. Length of stay (LOS);
3. Ventilator support, invasive (mechanical) and non-invasive ventilator support;
4. Severity score (simplified acute physiology score II (SAPS II) [10]) and organ dysfunction (sequential organ failure assessment score (SOFA) [11]): we defined severe organ dysfunctions as a SOFA score of 3 or 4; among daily SOFA scores, only the maximum was included in the analysis;
5. Comorbidity: we separated comorbidity in four categories (none, mild, moderate, and severe) based on the Charlson comorbidity index (CCI) [12];
6. Diagnostic groups: ICU admissions were allocated into one of thirteen different categories;
7. Short- and long-term survival (long-term defined as 1 year and longer): the standardized mortality ratio (SMR) was defined as the observed hospital mortality divided by the SAPS II estimated mortality; the SMR was analyzed for all patients and for each of the SAPS II admission categories;
8. Survival at follow-up; and
9. SAPS II admission categories, planned surgery, unplanned surgery, and medical reasons.

Survival was compared with a segment of the general population that was 80 years of age or older during 2000–2013, based on life tables from Statistics Norway.

The second part of the study included a prospective analysis of HRQOL. Patients alive at follow-up (16th of January 2014) were compared with a control group of 375 individuals matched for age, sex, and residence, which were randomly drawn from the National Registry. The HRQOL was assessed with EuroQol-5D (EQ-5D-3L) [13], a questionnaire sent by mail to ICU survivors and the control group at follow-up. EQ-5D has five dimensions, each with three response options. It also included a visual analog scale (EQ-VAS; Table 4). A reminder was sent to the non-responders after 1 month. ICU survivors were also contacted by phone. Informed consent was given by persons who answered the questionnaire.

We compared hospital survivors with hospital non-survivors and also compared the SAPS II admission categories. Information about end-of-life decision-making was retrospectively found for hospital non-survivors by searching through the individual patient files of their current hospital stay since such information was not entered in the ICU database. We only included statements which clearly used the terms withholding or withdrawal of ICU treatment.

The study was approved by the Regional Committee of Medical and Health Research Ethics in Central Norway (REC Central, 2013/1113).

### Statistics

The length of stay (LOS) and ventilator time are expressed in terms of medians and quartiles. Significance was tested with the Mann-Whitney U test/Kruskal-Wallis test. Other continuous variables are expressed as the mean with standard deviation (SD) and compared with the *t* test/analysis of variance (ANOVA). Qualitative and dichotomous data are reported as the percent of *n*, and they were compared with Pearson’s chi-square test/Fisher’s exact test or with the Mann-Whitney U test. Three separate Cox proportional hazard regression analyses were used to determine independent predictors of ICU mortality, hospital mortality, and 1-year mortality. The time factor was defined as the number of days from ICU admission, ICU discharge, and hospital discharge, respectively. All variables with a *p* value of <0.2 in a primary univariate analysis were included in the multivariate model, except for admission categories; admission categories were included even when the *p* value was >0.2 in the univariate analysis. ICU mortality was analyzed separately. Only ICU survivors were included in the analysis of hospital mortality. Only hospital survivors were included in the 1-year mortality analysis. The remaining variables were then tested separately in the models, and included if they were significant. Adjusted hazard ratios (HR) were calculated with 95 % confidence intervals (95 % CI). Kaplan-Meier curves were constructed from the three SAPS II admission categories. Another Kaplan-Meier curve was constructed to compare all patients to the general octogenarian population in Norway. An adjusted mortality rate was calculated by dividing the observed mortality rate by the expected mortality rate from an age- and gender-matched population. The adjusted mortality rate was calculated between 1 and 8 years after ICU admission. Patients who were alive at follow-up were censored. All statistical analyses were performed with SPSS 21.0 (SPSS Inc., Chicago, IL, USA). *P* values <0.05 were considered statistically significant.

## Results

**Fig. 1:**
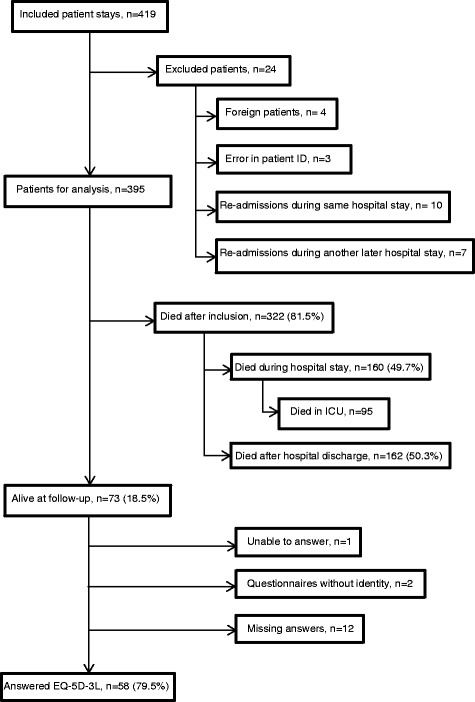
Data collection process

From 2000 to 2012, 402 patients ≥80 years were admitted to our ICU, with a total of 419 ICU stays. Re-admissions (during the same hospital stay (*n* = 10) and during another later hospital stay (*n* = 7)), non-Norwegian patients (*n* = 4), and admissions with errors in patient ID (*n* = 3) were omitted from the analysis. Thus, 395 patients were included in the current study (Fig. 1).

### Patient characteristics

#### Age and gender

At ICU admittance, the mean age was 83.8 years (range 80–101; median 83.1) and 61.0 % were males (Table 1). Males had longer median ICU-LOS (2.1 vs. 1.5 days, *p* = 0.006), a higher mean maximum SOFA score (8.3 vs. 7.0, *p* = 0.031), and severe circulatory failure more frequently (52.3 vs. 39.0 %, *p* = 0.010) than females.

**Table 1:** Differences in characteristics between hospital survivors and hospital non-survivors

	Total (*n* = 395)	Hospital survivors (*n* = 235)	Hospital non-survivors (*n* = 160)	*p* value
Age, mean ± SD	83.8 ± 2.9	83.5 ± 2.9	84.1 ± 2.8	0.049^a^
Male, %	61.0	60.9	61.3	0.511^b^
Length of stay (LOS), median (IQR)				
ICU-LOS	1.8 (0.9–3.9)	1.9 (1.0–4.3)	1.7 (0.8–3.2)	0.097^c^
Hospital LOS	11.3 (4.0–19.3)	14.2 (7.6–25.1)	5.5 (1.9–12.8)	<0.001^c^
Ventilator support				
Mechanical ventilator support, % (*n*)	61.3 (242)	51.9 (122)	75.0 (120)	<0.001b
Mechanical ventilator support time, median (IQR)	1.2 (0.5–3.3)	1.3 (0.5–3.8)	1.0 (0.4–3.0)	0.235^c^
Non-invasive ventilator support, % (*n*)	33.2 (131)	35.8 (84)	29.6 (47)	0.344^b^
Non-invasive ventilator support time, median (IQR)	1.5 (0.5–2.8)	1.6 (0.5–3.2)	1.3 (0.4–2.5)	0.164^c^
Severity score, mean ± SD				
SAPS II	44.3 ± 15.0 (*n* = 390)	40.6 ± 12.9 (*n* = 230)	49.5 ± 16.3 (*n* = 160)	<0.001^a^
Max. SOFA	7.8 ± 3.8 (*n* = 389)	6.7 ± 3.3 (*n* = 229)	9.5 ± 3.8 (*n* = 160)	<0.001^a^
Comorbidity				
Charlson comorbidity index, mean ± SD	2.6 ± 1.9 (*n* = 390)	2.7 ± 1.8 (*n* = 234)	2.5 ± 1.9 (*n* = 156)	0.389^a^
Charlson comorbidity index (CCI) categories, % (*n*)				0.602^b^
None (CCI 0)	12.3 (48)	11.1 (26)	14.1 (22)	
Mild (CCI 1–2)	40.8 (159)	39.3 (92)	42.9 (67)	
Moderate (CCI 3–4)	32.3 (126)	34.2 (80)	29.5 (46)	
Severe (CCI ≥5)	14.6 (57)	15.4 (36)	13.5 (21)	
Severe organ dysfunction, % (*n*)				
Respiration	66.3 (262)	62.6 (147)	71.9 (115)	0.034^b^
Circulation	47.1 (186)	38.7 (91)	59.4 (95)	<0.001^b^
Renal	28.1 (111)	20.9 (49)	38.8 (62)	<0.001^b^
CNS	26.1 (103)	18.7 (44)	36.9 (59)	<0.001^b^
Coagulation	9.9 (39)	9.8 (23)	10.0 (16)	0.537^b^
Liver	1.3 (5)	0.9 (2)	1.9 (3)	0.399^d^
Admission categories, % (*n*)				<0.001^b^
Planned surgery	12.7 (50)	17.9 (42)	5.0 (8)	
Unplanned surgery	53.9 (213)	49.8 (117)	60.0 (96)	
Medical reasons	33.4 (132)	32.3 (76)	35.0 (56)	
Diagnostic groups, % (*n*)				
Respiratory failure	28.1 (111)	31.1 (73)	23.8 (38)	0.112^b^
Circulatory failure	8.1 (32)	7.7 (18)	8.8 (14)	0.697^b^
Combined respiratory and circulatory failure	10.4 (41)	8.9 (21)	12.5 (20)	0.254^b^
Neurologic failure	10.1 (40)	9.4 (22)	11.3 (18)	0.541^b^
Isolated head injury	2.5 (10)	1.7 (4)	3.8 (6)	0.328^d^
Sepsis	8.9 (35)	7.2 (17)	11.3 (18)	0.168^b^
Gastroenterological failure	4.8 (19)	4.7 (11)	5.0 (8)	0.884^b^
Multiple organ failure	5.6 (22)	3.0 (7)	9.4 (15)	0.007^b^
Multitrauma without head injury	3.8 (15)	5.1 (12)	1.9 (3)	0.099^b^
Multitrauma with head injury	2.3 (9)	1.7 (4)	3.1 (5)	0.495^d^
Planned surgery	3.3 (13)	5.5 (13)	0.0 (0)	0.002^b^
Acute operation	6.6 (26)	7.2 (17)	5.6 (9)	0.527^b^
Unspecified	5.6 (22)	6.8 (16)	3.8 (6)	0.193^b^

*IQR* interquartile range, *SD* standard deviation, *CI* confidence interval, *ICU* intensive care unit, *SAPS II* simplified acute physiology score II, *SOFA* sequential organ failure assessment, *CCI* Charlson comorbidity index

^a^Independent *t* test

^b^Pearson’s chi-square

^c^Mann-Whitney U test

^d^Fisher’s exact test

#### Length of stay

The overall median ICU-LOS and hospital-LOS were 1.8 and 11.3 days, respectively. The median LOS for ICU non-survivors was 1.3 days (see Table 1). Among all patients, 26.8 % stayed less than 1 day in the ICU.

#### Ventilator support

Of 395 patients, 61.3 % received mechanical ventilator support for a median time of 1.2 days. Of the hospital non-survivors, 75.0 % (*n* = 120) received mechanical ventilator support (Table 1). A fraction of 69.2 % (*n* = 83) of these 120 patients had life-sustaining treatment limitation decisions.

#### Severity scores and severe organ dysfunction

Overall, the mean SAPS II and mean maximal SOFA scores were 44.3 and 7.8, respectively. Hospital non-survivors had a mean SAPS II of 49.5 and a mean maximal SOFA score of 9.5 (Table 1). All patients with maximal SOFA scores ≥17 died in the ICU; all those with scores ≥16 died during the hospital stay.

#### Comorbidity

Overall mean Charlson comorbidity index was 2.6. Patients admitted for planned surgery showed the highest index score among the admission categories (3.2). Only 12.3 % of the patients had no preexisting comorbidity (see Table 1 and 2).

**Table 2:** Characteristics of SAPS II admission categories

	Planned surgery (*n* = 50)	Unplanned surgery (*n* = 213)	Medical reasons (*n* = 132)	Total (*n* = 395)	*p* value
Age, mean ± SD	83.5 ± 2.7	84.0 ± 2.7	83.5 ± 3.2	83.8 ± 2.9	0.217^a^
Male, %	64.0	60.6	60.6	61.0	0.889^b^
Length of stay (LOS), median (IQR)					
ICU-LOS	2.0 (1.0–4.4)	2.2 (1.0–5.0)	1.2 (0.6–2.6)	1.8 (0.9–3.9)	<0.001^b^
Hospital LOS	15.1 (10.2–26.2)	12.9 (4.3–20.6)	6.5 (2.0–14.3)	11.3 (4.0–19.3)	<0.001^b^
Ventilator support					
Mechanical ventilator support, % (*n*)	48.0 (24)	69.5 (148)	53.0 (70)	61.3 (242)	0.001^b^
Mechanical ventilator support time, median (IQR)	1.1 (0.4–3.7)	1.3 (0.5–3.8)	0.9 (0.3–2.0)	1.2 (0.5–3.3)	0.050^b^
Non-invasive ventilator support, % (*n*)	44.0 (22)	33.8 (72)	28.0 (37)	29.6 (47)	0.119^b^
Non-invasive ventilator support time, median (IQR)	1.2 (0.5–2.6)	2.0 (0.6–3.0)	1.0 (0.3–2.0)	1.3 (0.4–2.5)	0.056^b^
Severity score, mean ± SD					
SAPS II	39.0 ± 13.2 (*n* = 47)	44.6 ± 14.8 (*n* = 212)	45.6 ± 15.6 (*n* = 131)	44.3 ± 15.0 (*n* = 390)	0.030^a^
Max. SOFA	6.3 ± 4.1 (*n* = 47)	8.3 ± 3.6 (*n* = 212)	7.5 ± 3.8 (*n* = 130)	7.8 ± 3.8 (*n* = 389)	0.002^a^
Comorbidity					
Charlson comorbidity index, mean ± SD	3.2 ± 1.8 (*n* = 50)	2.5 ± 2.0 (*n* = 209)	2.5 ± 1.7 (*n* = 131)	2.6 ± 1.9 (*n* = 390)	0.050^a^
Charlson comorbidity index (CCI) categories, % (*n*)					
None (CCI 0)	4.0 (2)	13.4 (28)	13.7 (18)	12.3 (48)	0.159^b^
Mild (CCI 1–2)	34.0 (17)	43.1 (90)	39.7 (52)	40.8 (159)	0.436^b^
Moderate (CCI 3–4)	46.0 (23)	26.3 (55)	36.6 (48)	32.3 (126)	0.009^b^
Severe (CCI ≥5)	16.0 (8)	17.2 (36)	9.9 (13)	14.6 (57)	0.163^b^
Severe organ dysfunction, % (*n*)					
Respiration	68.0 (34)	71.8 (153)	56.8 (75)	66.3 (262)	0.016^b^
Circulation	38.0 (19)	53.1 (113)	40.9 (54)	47.1 (186)	0.035^b^
Renal	30.0 (15)	28.6 (61)	26.5 (35)	28.1 (111)	0.868^b^
CNS	20.0 (10)	23.5 (50)	32.6 (43)	26.1 (103)	0.100^b^
Coagulation	16.0 (8)	8.5 (18)	9.8 (13)	9.9 (39)	0.273^b^
Liver	2.0 (1)	0.0 (0)	3.0 (4)	1.3 (5)	0.034^d^
Survival, % (*n*)					
ICU	90.0 (45)	74.2 (158)	73.5 (97)	75.9 (300)	0.045^b^
Hospital	84.0 (42)	54.9 (117)	57.6 (76)	59.5 (235)	0.001^b^
30 days	86.0 (43)	51.2 (109)	54.5 (72)	56.7 (224)	<0.001^b^
90 days	82.0 (41)	44.6 (95)	50.8 (67)	51.4 (203)	<0.001^b^
180 days	74.0 (37)	40.4 (86)	47.7 (63)	47.1 (186)	<0.001^b^
1 year	68.0 (34)	37.1 (79)	40.2 (53)	42.0 (166)	<0.001^b^
2 years	59.9 (28)	33.1 (64)	33.6 (39)	36.6 (130)	0.001^b^
3 years	48.4 (15)	27.8 (50)	29.9 (32)	31.2 (96)	0.088^b^
5 years	32.8 (7)	18.6 (28)	23.7 (22)	22.2 (55)	0.290^b^
Diagnostic groups, % (*n*)					
Respiratory failure	44.0 (22)	24.4 (52)	28.0 (37)	28.1 (111)	0.021^b^
Circulatory failure	8.0 (4)	8.5 (18)	7.6 (10)	8.1 (32)	0.959^b^
Combined respiratory and circulatory failure	8.0 (4)	12.7 (27)	7.6 (10)	10.4 (41)	0.270^b^
Neurologic failure	2.0 (1)	7.0 (15)	18.2 (24)	10.1 (40)	<0.001^b^
Isolated head injury	0.0 (0)	1.9 (4)	4.5 (6)	2.5 (10)	0.165^d^
Sepsis	4.0 (2)	8.9 (19)	10.6 (14)	8.9 (35)	0.308^d^
Gastroenterological failure	0 (0)	5.6 (12)	5.3 (7)	4.8 (19)	0.276^d^
Multiple organ failure	4.0 (2)	7.5 (16)	3.0 (4)	5.6 (22)	0.205^d^
Multitrauma without head injury	0.0 (0)	5.6 (12)	2.3 (3)	3.8 (15)	0.107^d^
Multitrauma with head injury	0.0 (0)	2.3 (5)	3.0 (4)	2.3 (9)	0.638^d^
Planned surgery	18.0 (9)	1.4 (3)	0.8 (1)	3.3 (13)	<0.001^d^
Acute operation	2.0 (1)	10.3 (22)	2.3 (3)	6.6 (26)	0.004^d^
Unspecified	10.0 (5)	3.8 (8)	6.8 (9)	5.6 (22)	0.150^d^
SMR (95 % CI)	0.55 (0.28–1.11) (*n* = 47)	1.15 (0.94–1.40) (*n* = 212)	1.05 (0.81–1.37) (*n* = 131)	1.06 (0.90–1.23) (*n* = 390)	

Survival times were derived from the life table method

*IQR* interquartile range, *SD* standard deviation, *CI* confidence interval, *ICU* intensive care unit, *SAPS II* simplified acute physiology score II, *SOFA* sequential organ failure assessment, *CCI* Charlson comorbidity index, *SMR* standardized mortality ratio

^a^With ANOVA (analysis of variance)

^b^Pearson’s chi-square test

^c^With Kruskal-Wallis test

^d^Fisher’s exact test

#### Diagnostic groups

The most frequent cause for ICU admission was respiratory failure (28 %). Respiratory failure was most common in the planned surgery group (44 %; Table 2).

### Survival and predictors of mortality

#### Short-term survival

The overall ICU and hospital survival were 75.9 and 59.5 %, respectively. Of the ICU non-survivors, 63.2 % died within 2 days after ICU admission (*n* = 60), and 68.3 % of these patients had life-sustaining treatment (LST) limitations ((*n* = 41); withholding 60.0 % and withdrawal 51.7 %). The SMR was 1.06, with large differences between the planned surgery (0.55) and unplanned surgery (1.15) groups. For survival at 30, 90, and 180 days, see Table 2.

Predictors of high ICU mortality were age, mechanical ventilator support, SAPS II, maximum SOFA, and multitrauma with head injury. Increased hospital mortality was predicted by an unplanned surgical admission (Table 3).

**Table 3:** Predictors of mortality in the ICU, in hospital, and at 1 year after admission

	ICU mortality (*n* = 389)	Hospital mortality for ICU survivors (*n* = 294)	1-year mortality for hospital survivors (*n* = 230)
	Adjusted HR (95 % CI)	Adjusted HR (95 % CI)	Adjusted HR (95 % CI)
Age, years	1.10 (1.03–1.18)^a^		
Male			
Ventilator support			
Mechanical ventilator support	1.99 (1.10–3.60)^a^	1.40 (0.81–2.43)	
Non-invasive ventilator support	0.87 (0.51–1.49)		
Severity score, mean			
SAPS II	1.03 (1.01–1.04)^a^	1.01 (0.99–1.03)	1.01 (0.99–1.03)
Max. SOFA	1.20 (1.10–1.31)^a^	1.03 (0.95–1.12)	
Comorbidity			
None (CCI 0)	1.00		1.00
Mild (CCI 1–2)	0.68 (0.35–1.30)		1.02 (0.43–2.46)
Moderate (CCI 3–4)	0.53 (0.25–1.11)		1.06 (0.42–2.65)
Severe (CCI ≥ 5)	0.53 (0.23–1.25)		2.09 (0.99–5.39)
Severe organ dysfunction			
Respiration	1.05 (0.55–1.97)		
Circulation	0.76 (0.39–1.48)		
Renal	1.50 (0.88–2.54)		
CNS	1.19 (0.71–1.99)		
Coagulation			
Liver			
Admission categories			
Planned surgery	1.00	1.00	1.00
Unplanned surgery	1.40 (0.54–3.65)	3.46 (1.06–11.24)^a^	2.02 (0.88–4.64)
Medical reasons	2.11 (0.80–5.58)	3.17 (0.94–10.76)	1.97 (0.83–4.70)
Diagnostic groups			
Respiratory failure	1.03 (0.55–1.90)		1.86 (1.13–3.07)^a^
Circulatory failure			
Combined respiratory and circulatory failure			
Neurologic failure		1.67 (0.86–3.25)	
Isolated head injury	1.56 (0.58–4.18)		9.12 (2.44–34.14)^a^
Sepsis	1.20 (0.63–2.69)		
Gastroenterological failure			
Multiple organ failure	1.27 (0.60–2.69)	1.67 (0.64–4.31)	
Multitrauma without head injury			
Multitrauma with head injury	2.99 (1.04–8.60)^a^		
Planned surgery			
Acute operation			
Unspecified			

*ICU* intensive care unit, *SAPS II* simplified acute physiology score II, *SOFA* sequential organ failure assessment, *HR* hazard ratio, *CI* confidence interval, *CCI* Charlson comorbidity index

^a^Significant differences

#### Long-term survival (1 year and longer)

**Fig. 2:**
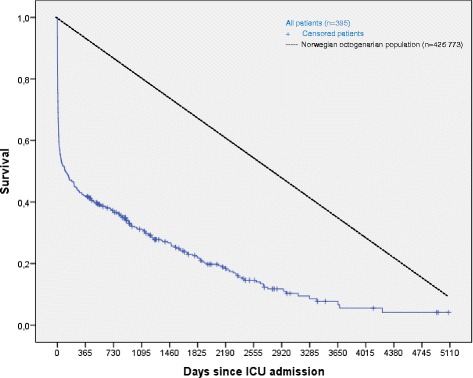
Kaplan-Meier survival curve for all patients (*solid blue line*) compared to the Norwegian octogenarian population (*dashed black line*) in 2000–2013

The overall 1- and 2-year survival rates were 42.0 and 36.6 %, respectively. After 5 years, 22.2 % of all patients remained alive. A comparison between patients (*n* = 395) and the general population greater than 80 years old in Norway (*n* = 426 773) showed excess mortality among patients in the first year, with an adjusted mortality rate of 6.35 (95 % CI 5.58–7.23). After the first year, the survival rates were similar between groups; patients had an adjusted mortality rate during the second year of 1.34 (95 % CI 0.86–2.07; Fig. 2). Among patients alive after 1 year, the mean survival time, starting from the 1-year point, was 5.1 years.

Respiratory failure and isolated head injury were independent predictors of 1-year mortality (Table 3).

#### Survival at follow-up

At follow-up (January 2014), 322 (81.5 %) patients had died, including 160 during the hospital stay and 162 after hospital discharge. Seventy-three patients (18.5 %) survived, with a mean age of 86.9 years at follow-up. The median time from hospital discharge to follow-up was 3.3 years (range 1–13.8 years; Fig. 1). The survivors at follow-up (*n* = 73) had, compared to hospital survivors not alive at follow-up (*n* = 162), similar ICU-LOS (1.9 vs. 1.8 days; *p* = 0.465), fraction of ventilator support (52.1 vs. 51.9 %; *p* = 0.977), severity of illness (SAPS II 43.2 vs. 39.4, *p* = 0.658; max. SOFA 6.5 vs. 6.7, *p* = 0.313), and comorbidity (Charlson comorbidity index 2.4 vs. 2.8, *p* = 0.156; Additional file 1: Table S1). However, hospital survivors not alive at follow-up had a lower median survival after hospital discharge (3.1 years), compared to the follow-up of 3.4 years in survivors.

### SAPS II admission categories

**Fig. 3:**
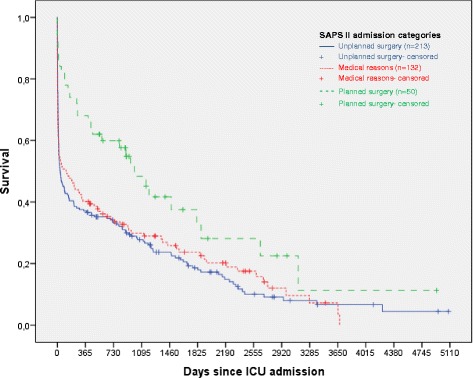
Kaplan-Meier survival curves of SAPS II admission categories

Patients admitted for planned surgery had significantly higher survival rates than those admitted for medical reasons and unplanned surgery up to 3 years after ICU admittance (Table 2). The median survival times were 33.4 months (95 % CI 21.2–45.6) for planned surgery, 1.2 months (95 % CI 0.0–2.7) for unplanned surgery, and 2.7 months (95 % CI 0.0–9.1) for medical admissions (Fig. 3).

### Health-related quality of life

The EQ-5D questionnaire was sent to the 73 patients who were alive at follow-up. The response rate was 83.6 % (*n* = 61), but one questionnaire was incomplete, and two questionnaires had no patient identity. Fourteen patients responded to the questionnaire by telephone. The response rate in the control group was 47.7 % (179/375), constituting 2.5 controls per survivor at follow-up. HRQOL was similar between patients and the general population and among the admission categories (Table 4).

**Table 4:** Comparison of frequency distributions (profiles) of the EQ-5D-3L for patient and control groups

Variable	Total patients (*n* = 58)	Control group (*n* = 179)	*p* value
Age, years, mean ± SD	87.4 ± 4.0	86.7 ± 4.4	0.265^a^
Male, % (*n*)	69.0 (40)	66.5 (119)	0.726^b^
Mobility, % (*n*)			0.504^c^
No problem	41.4 (24)	43.6 (78)	
Some problems	51.7 (30)	54.7 (98)	
Confined to bed	6.9 (4)	1.7 (3)	
Self-care, % (*n*)			0.957^c^
No problem	75.9 (44)	74.9 (134)	
Some problems	15.5 (9)	21.8 (39)	
Unable to	8.6 (5)	3.4 (6)	
Usual activities, % (*n*)			0.237^c^
No problem	43.1 (25)	49.7 (89)	
Some problems	41.4 (24)	41.3 (74)	
Unable to	15.5 (9)	8.9 (16)	
Pain and discomfort, % (*n*)			0.229^c^
None	43.1 (25)	34.6 (62)	
Moderate	51.7 (30)	58.1 (104)	
Extreme	5.2 (3)	7.3 (13)	
Anxiety and depression, % (*n*)			0.258^c^
None	77.6 (45)	69.8 (125)	
Moderate	20.7 (12)	27.9 (50)	
Extreme	1.7 (1)	2.2 (4)	
EQ index, mean ± SD	0.71 ± 0.28	0.73 ± 0.23	0.924^c^
EQ VAS, mean ± SD	63.9 ± 20.3 (*n* = 53)	67.7 ± 22.0 (*n* = 170)	0.219^c^

*SD* standard deviation, *EQ* EuroQol, *VAS* visual analog scale (range 0–100)

^a^Independent *t* test

^b^Pearson’s chi-square

^c^Mann-Whitney U test

### Life-sustaining treatment limitation in hospital non-survivors

Of the ICU non-survivors, 70.5 % (*n* = 67) had treatment-limitation decisions; withholding 68.4 % (*n* = 65) and withdrawal 51.6 % (*n* = 49). The majority of these LST limitation decisions were taken within the first 2 days after ICU admission (61.2 % (*n* = 41)). Post ICU 72.3 % (*n* = 47) of the hospital non-survivors had treatment-limitation decisions; withholding 72.3 % (*n* = 47) and withdrawal 32.3 % (*n* = 21). We lack information on LST decisions in six ICU non-survivors and two ICU survivors.

## Discussion

This study establishes three major results. First, patients who survived the first year after ICU admittance showed long-term survival rates similar to those of the normal Norwegian octogenarian population. The HRQOL of long-time survivors was comparable to that of an age- and sex-matched general population group. Second, the planned surgery group exhibited higher survival rates than the medical and unplanned surgery groups up to 3 years after ICU admittance. However, at follow-up, HRQOL did not differ among these three groups. Third, high ICU mortality was predicted by age, mechanical ventilator support, SAPS II, maximum SOFA score, and multitrauma with head injury. High hospital mortality was predicted by an unplanned surgical admission. Respiratory failure and isolated head injury were independent predictors of 1-year mortality. The majority of the ICU non-survivors died within 2 days, where most of these had life-sustaining treatment (LST) limitations. Almost three quarters of the hospital non-survivors had treatment-limitation decisions.

Our finding of age as an independent predictor of ICU mortality contrasts with several previous studies [14, 15]. Conflicting results about the impact of age on outcome for older patients in the ICU may be explained by variations in adjustments for severity and comorbidities among different studies. Moreover, in some institutions, older individuals may have been denied ICU admission, based on advanced age [16]. In addition, treatment is often withheld for older ICU patients with severe comorbidity [17]. In our study, advanced age may also have influenced preferences in decision-making among patients, relatives, or caregivers. The influence of age on mortality must be adjusted for severity of illness.

In general, ICU length of stay is short in Scandinavian countries [6, 7, 18]. One explanation can be the low availability of ICU beds compared to many other European countries [3, 19]. Also, octogenarians are in general found to have shorter ICU stays than younger patients [6, 20, 21]. This is probably reflected in our study. The overall median ICU-LOS was 1.8 days, which was 3.2–4.2 days shorter than that reported in recent French studies [8, 9]. Also, our ICU and hospital mortality rates were lower than reported in those studies. These findings might be explained by differences in “case-mix” within the SAPS II admission categories, where the French studies included mostly medical cases. However, our medical group had significantly shorter ICU stays (median 1.2 days) than the unplanned surgery group. This finding could not be explained by differences in mortalities or SAPS II scores. Even though ICU-LOS is short in our study, the mean SAPS II scores and mechanical ventilator support rates are comparable to other octogenarian cohort studies [8, 9, 14, 21–23]. In general, our survivors had longer ICU stays than non-survivors, due to death shortly after ICU admittance (63.2 % within 2 days). The large proportion of LST limitations among ICU non-survivors during the first 2 days after ICU admission may contribute to the short length of stay. Our data could indicate that ICU physicians limit the intensity of life-sustaining treatment if there is no improvement in the condition of the octogenarian patient within the first 2 days after ICU admission. Although we lack data on triage decisions prior to ICU admission, we might speculate that a more thorough pre-ICU triage process could have decreased the rather high fraction of LST limitation decisions by rejecting patients who probably would not benefit from ICU treatment.

After the first year, we found our ICU patients to have survival rates similar to those of the general octogenarian population. Interestingly, Roch et al. found a similar trend after 2 years [9]. The low 1-year survival rate may indicate that many aged patients did not benefit from ICU treatment. Therefore, we need better predictors to determine which patients are likely to gain long-term benefit from ICU treatment. Several studies have reported prognostic factors for short- and long-term mortality among older individuals [14, 17, 24]. In general, short-term mortality is most frequently predicted by severity scores and the number of organ failures [17]. Commonly used prognostic models for aged patients in the ICU lack calibration. Nevertheless, our study showed that severity scores were good predictors for ICU mortality, in addition to age, mechanical ventilator support, and multitrauma with head injury. One study developed a prognostic model for predicting in-hospital mortality in older patients in the ICU, and found low Glacow coma scale (GCS) scores to be strongly related to short-term mortality [24]. Several other studies have reported that brain injury is associated with poor outcomes in older patients [25, 26]. Comorbidity is also found to be a predictor of long-term survival in some octogenarian ICU studies [9, 14]. However, these studies used the McCabe classification, where comorbidity is based on the presence of underlying fatal diseases. In our study, we found no association between long-term mortality and comorbidity, using the Charlson comorbidity index. This is supported in other studies [5, 27], also using Charlson comorbidity index. The regression analysis of ICU mortality showed decreasing hazard of death with increasing comorbidity. This was probably mainly due to admission of patients with no comorbidity who suffered severe trauma and bleeding events, with high mortality. We might speculate that the admission policy of these patients was more liberal due to lack of comorbidity, even if the prospects of survival was low, compared to patients with higher comorbidity. In our opinion, comorbidity is not a very useful predictor for ICU mortality in general nor for the elderly population.

To our knowledge, this study is the first to report HRQOL in older patients over a 13-year post-ICU follow-up. We found similar HRQOLs in ICU survivors and the general Norwegian octogenarian population at follow-up. Other recent studies on HRQOL in older ICU survivors have reported impaired physical function [9, 28]. De Rooij et al. found that patients had more problems with usual activities and lower mean EQ VAS scores than the general British population [29]. In contrast, Tabah et al. reported a similar HRQOL, or better in some domains, compared to a matched general population [8]. Good HRQOL perceptions, despite physical impairment, could be due to lower expectations of life after critical illness. However, HRQOL evaluations may be prone to selection bias, because responders may represent healthier patients. Our study revealed that non-responders and responders had similar severity scores and similar fractions of severe organ failures. But non-responders were evaluated at slightly longer times after hospital discharge (median 4.6 vs. 3.3 years; *p* = 0.350). Patients alive after 1 year had a mean further survival time of 5.1 years. Furthermore, survivors at follow-up had longer time to follow-up compared to the median survival in hospital survivors not alive at follow-up. Nevertheless, these groups were otherwise comparable, and we can speculate that hospital survivors no longer alive had about the same HRQOL as survivors at follow-up, at least for much of the time left (Additional file 1: Table S1).

Very few studies have reported outcomes for aged patients in different SAPS II admission categories. De Rooij et al. reported higher short- and long-term survival in patients admitted for planned surgery compared to those admitted for medical reasons and unplanned surgery, with a mean follow-up of 3.6 years [22]. Our results supported that finding, but only up to 3 years after ICU admittance. Thereafter, long-term survival was similar among the groups. We also found that an unplanned surgery admission could predict high hospital mortality in ICU survivors.

### Limitations

This study has several limitations. First, it was partly a retrospective study and clinical data were confined to those registered in the ICU database. Thus, we had no information about triage decisions made before ICU admission. Variability in these decisions may influence the results [30–32]. Second, the long inclusion period could contain changes in admission policy and medical practice. However, the catchment area and basic functions of the hospital remained the same during the study period, with a slowly growing population and all medical services except organ transplant surgery offered. There were no large changes in practice or organizational changes in the ICU during the study period. Third, due to our single-center study design, the group sizes were relatively small. In particular, the number of patients for HRQOL assessment was limited (*n* = 73); this is common in single-center studies of aged ICU populations. Furthermore, the HRQOLs of different groups were evaluated at different follow-up times. Nevertheless, every patient was followed-up after at least 1 year, the recommended minimum [33]. Furthermore, the high response rate for the EQ-5D questionnaire (*n* = 58, 79.5 %) provided valuable HRQOL information among older, long-term ICU survivors in Norway, particularly compared to the age- and gender-matched control group. We evaluated HRQOL once in each patient; thus, we did not study changes in HRQOL over time. Ideally, a baseline measurement should be made before the ICU stay. Finally, we had no information on living status or cognitive functions.

## Conclusions

One-year survival was 42.0 %, with further survival comparable to the general octogenarian population. HRQOL in survivors was comparable with an age- and sex-matched general population, with a follow-up of 1–13.8 years. Up to 3 years after ICU admittance, patients admitted for planned surgery had better short- and long-term outcomes than those admitted for medical reasons and unplanned surgery. The majority of the ICU non-survivors died within 2 days, and most of these had life-sustaining treatment (LST) limitations. Almost three quarters of the hospital non-survivors had treatment-limitation decisions. Our results indicate that older ICU patients have poor short-term outcomes due to high mortalities, but good long-term outcomes in those who survive beyond 1 year. Predictors identified in this study may facilitate triage decisions in older patients regarding ICU treatment. Future research should focus on improving prognostic models for aged patients.

### Key messages

- One-year survival was 42.0 %; thereafter, survival was comparable to that of the general octogenarian population.
- HRQOL in our survivors at follow-up (*n* = 58) was comparable with an age- and gender-matched general population (*n* = 179), for a follow-up of 1–13.8 years.
- Patients admitted for planned surgery had better short- and long-term survival rates than those admitted for medical reasons or unplanned surgeries for three years after ICU admittance.
- The majority of the ICU non-survivors died within 2 days (63.2 %), and most of these had life-sustaining treatment (LST) limitations (68.3 %).

## Additional file

Additional file 1: Table S1. Differences in characteristics between hospital survivors not alive at follow-up (*n* = 162) and survivors at follow-up (*n* = 73).

## Abbreviations

HRQOL: health-related quality of life
ICU: intensive care unit
LOS: length of stay
SAPS II: simplified acute physiology score II
SOFA: sequential organ failure assessment score
CCI: Charlson comorbidity index
SMR: standardized mortality ratio
SD: standard deviation
CI: confidence interval
IQR: interquartile range
ANOVA: analysis of variance
HR: hazard ratio
LST: life-sustaining
