# Supplementary material for: Long-term survival and quality of life after intensive care for patients 80 years of age or older
Source: Ann Intensive Care. 2015 Jun 3;5:13. doi: 10.1186/s13613-015-0053-0 (PMC4456598; doi:10.1186/s13613-015-0053-0)
Supplement: Additional file 1: Table S1. — Differences in characteristics between hospital survivors not alive at follow-up (n = 162) and survivors at follow-up (n = 73). [file 13613_2015_53_MOESM1_ESM.docx]

Table S1. Differences in characteristics between hospital survivors not alive at follow-up (*n* = 162) and survivors at follow-up (*n* = 73)

|  | Hospital survivors (*n* = 235) | Hospital survivors not alive at follow-up (*n* = 162) | Survivors at follow-up (*n* = 73) | *p*-value |
| --- | --- | --- | --- | --- |
| **Age, mean ± SD**  **Age (follow-up), mean ± SD** | 83.5 ± 2.9 | 83.7 ± 3.1 | 83.2 ± 2.6  86.9 ± 4.2 | 0.087^a^ |
| **Male, %** | 60.9 | 59.3 | 64.4 | 0.456^b^ |
| **Length of stay (LOS), median (IQR)** |  |  |  |  |
| ICU-LOS | 1.9 (1.0-4.3) | 1.8 (0.9-4.4) | 1.9 (1.1-4.3) | 0.465^c^ |
| Hospital LOS | 14.2 (7.6-25.1) | 15.1 (8.0-25.8) | 13.8 (6.7-21.9) | 0.109^c^ |
| **Ventilator support** |  |  |  |  |
| Mechanical ventilator support, % (*n*) | 51.9 (122) | 51.9 (84) | 52.1 (38) | 0.977^b^ |
| Mechanical ventilator time, median (IQR) | 1.3 (0.5-3.8) | 1.3 (0.5-4.1) | 1.4 (0.4-3.8) | 0.949^c^ |
| Non-invasive ventilator support, % (*n*) | 35.8 (84) | 38.3 (62) | 30.1 (22) | 0.229^b^ |
| Non-invasive ventilator support time, median (IQR) | 1.6 (0.5-3.2) | 2.0 (0.9-3.6) | 0.5 (0.3-2.4) | 0.010^c^ |
| **Severity score, mean ± SD** |  |  |  |  |
| SAPS II | 40.6 ± 12.9 (*n*=230) | 39.4 ± 12.8 (*n*=158) | 43.2 ± 12.6 (*n*=72) | 0.658^a^ |
| Max. SOFA | 6.7 ± 3.3 (*n*=229) | 6.7 ± 3.2 (*n*=157) | 6.5± 3.5 (*n*=72) | 0.313^a^ |
| **Comorbidity** |  |  |  |  |
| Charlson comorbidity index (CCI) score, mean ± SD | 2.7 ± 1.8 (*n*=234) | 2.8 ± 1.9 (*n*=161) | 2.4 ± 1.7 (*n*=73) | 0.156^c^ |
| Charlson comorbidity index (CCI) categories, % (n) |  |  |  | 0.007^b^ |
| None (CCI 0) | 11.1 (26) | 8.7 (14) | 16.4 (12) |  |
| Mild (CCI 1-2) | 39.3 (92) | 41.0 (66) | 35.6 (26) |  |
| Moderate (CCI 3-4) | 34.2 (80) | 30.4 (49) | 42.5 (31) |  |
| Severe (CCI ≥ 5) | 15.4 (57) | 19.9 (32) | 5.5 (4) |  |
| **Severe organ dysfunction, %** |  |  |  |  |
| Respiration | 62.6 (147) | 63.6 (103) | 60.3 (44) | 0.628^b^ |
| Circulation | 38.7 (91) | 39.5 (64) | 37.0 (27) | 0.714^b^ |
| Renal | 20.9 (49) | 15.4 (25) | 32.9 (24) | 0.002^b^ |
| CNS | 18.7 (44) | 16.0 (26) | 24.7 (18) | 0.117^b^ |
| Coagulation | 9.8 (23) | 7.4 (12) | 15.1 (11) | 0.067^b^ |
| Liver | 0.9 (2) | 1.2 (2) | 0.0 (0) | 0.340^d^ |
| **Admission categories, %** |  |  |  | 0.078^b^ |
| Planned surgery | 17.9 (42) | 14.2 (23) | 26.0 (19) |  |
| Unplanned surgery | 49.8 (117) | 53.1 (86) | 42.5 (31) |  |
| Medical reasons | 32.3 (76) | 32.7 (53) | 31.5 (23) |  |
| **Diagnostic groups, %** |  |  |  |  |
| Respiratory failure | 31.1 (73) | 32.7 (53) | 27.4 (20) | 0.415^b^ |
| Circulatory failure | 7.7 (18) | 8.0 (13) | 6.8 (5) | 0.754^b^ |
| Combined respiratory and circulatory failure | 8.9 (21) | 10.5 (17) | 5.5 (4) | 0.212^b^ |
| Neurologic failure | 9.4 (22) | 8.6 (14) | 11.0 (8) | 0.573^b^ |
| Isolated head injury | 1.7 (4) | 2.5 (4) | 0.0 (0) | 0.313^d^ |
| Sepsis | 7.2 (17) | 8.0 (13) | 5.5 (4) | 0.486^b^ |
| Gastroenterological failure | 4.7 (11) | 4.3 (7) | 5.5 (4) | 0.743^d^ |
| Multiple organ failure | 3.0 (7) | 3.7 (6) | 1.4 (1) | 0.440^d^ |
| Multitrauma without head injury | 5.1 (12) | 4.3 (7) | 6.8 (5) | 0.523^d^ |
| Multitrauma with head injury | 1.7 (4) | 1.2 (2) | 2.7 (2) | 0.590^d^ |
| Planned surgery | 5.5 (13) | 3.7 (6) | 9.6 (7) | 0.118^d^ |
| Acute operation | 7.2 (17) | 8.0 (13) | 5.5 (4) | 0.486^b^ |
| Unspecified | 6.8 (16) | 4.3 (7) | 12.3 (9) | 0.046^b^ |

IQR, interquartile range; SD, standard deviation; CI, confidence interval; ICU, intensive care unit; SAPS II, Simplified Acute Physiology Score II; SOFA, Sequential Organ Failure Assessment; CCI, Charlson comorbidity index

Significance tested with: ^a^ Independent t-test; ^b^ Pearson Chi-Square; ^c^ Mann Whitney U test; ^d^ Fisher’s exact test
